# 169. The Resurgence of *Candida auris* in California during the Novel Coronavirus (COVID-19) Pandemic, May 2020–May 2021

**DOI:** 10.1093/ofid/ofab466.169

**Published:** 2021-12-04

**Authors:** Tisha Mitsunaga, Diana Holden, Ellora Karmarkar, Idamae Kennedy, Teresa Nelson, Vikram Haridass, Alissa Dratch, Kathleen O’Donnell, Sandeep Bhaurla, Kelsey OYong, Anthony Clarke, Eric Takiguchi, Leslie Baldwin, Jennifer Nguyen, Kiran Bhurtyal, Alma Gomez, Kelli A Clark, Jessica R Batres, Scarlett Romo, Grace Kang, Mara Rauhauser, Emily C Schneider, Raymond Y Chinn, Barbara Cole, Michael Sequeira, Erin Gustafson, Emily Holman, Zachary Rubin, Matthew Zahn, Erin Epson

**Affiliations:** 1 California Department of Public Health, Richmond, CA; 2 Centers for Disease Control and Prevention, Richmond, CA; 3 Orange County Health Care Agency, Santa Ana, California; 4 Los Angeles County Department of Public Health, Los Angeles, CA; 5 Riverside University Health System – Public Health, Riverside, California; 6 San Bernardino County Department of Public Health, San Bernardino, California; 7 County of San Diego, Epidemiology & Immunization Services Branch, San Diego, California; 8 San Diego County Health & Human Services Agency, San Diego, California; 9 Washington State Department of Health, Shoreline, Washington; 10 County of San Diego, Health and Human Services Agency, San Diego, California; 11 Long Beach Department of Health and Human Services, Long Beach, California; 12 Orange County Department of Health, Irvine, California

## Abstract

**Background:**

In February 2019, California (CA) experienced its first *C. auris* outbreak in Orange County (OC). The CA Department of Public Health (CDPH) and OC with the Centers for Disease Control and Prevention (CDC), mounted a successful containment response; by November 2019, cases were limited to low-level spread in OC long-term acute care hospitals (LTACH).

In May 2020, *C. auris* cases began to surge in OC, followed by extensive spread in six other southern CA local health jurisdictions (LHJ). CDPH with LHJ and CDC, initiated an aggressive, interjurisdictional containment response.

**Methods:**

We carried out response and preventive point prevalence surveys (PPS), onsite infection prevention and control (IPC) assessments, and in-service trainings at outbreak and interconnected hospitals and skilled nursing facilities in six LHJ. Other regional activities included: epidemiologic investigation, contact and discharge tracking and screening; increasing laboratory testing capacity; screening patients admitted to and from LTACH; statewide healthcare facility (HCF) education and outreach; sending regional outbreak HCF lists to all HCF; and biweekly state-LHJ coordination calls. The Antibiotic Resistance (AR) Lab Network supported testing.

**Results:**

From May 2020—May 2021, we conducted screening at 226 HCF, and identified 1192 cases at 93 HCF, mostly through screening (n=1109, 93%) and at LTACH (n=906, 76%); we identified 113 (10%) cases at ACH, including 35 (31%) in COVID-19-burdened units. Cases peaked in August 2020 (n=93) and February 2021 (n=191) and have since declined, with *C. auris* resurgence mirroring COVID-19 incidence.

We conducted 98 onsite IPC assessments, and identified multiple, improper IPC practices which had been implemented in response to COVID-19, including double-gloving and -gowning, extended use of gowns and gloves outside patient rooms, and cohorting according to COVID-19 status only.

Figure 1. *C. auris* and COVID-19 Cases in California through May 2021, and *C. auris* Cases by Local Health Jurisdiction (LHJ) May 2020–May 2021

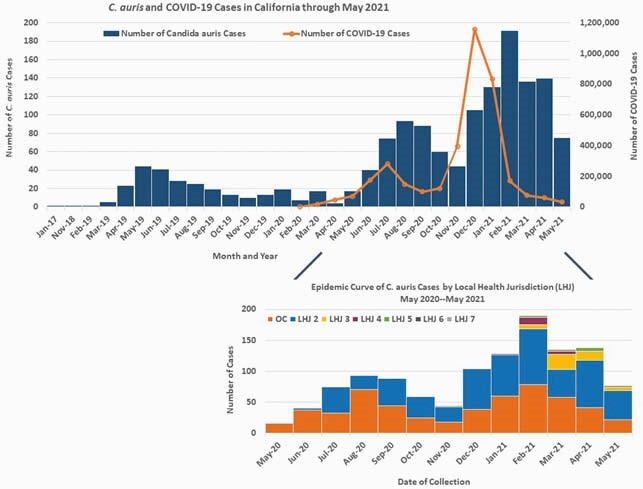

Table 1. By Facility Type: Colonization Testing May 2020–May 2021, and Total Case Counts before and from May 2020

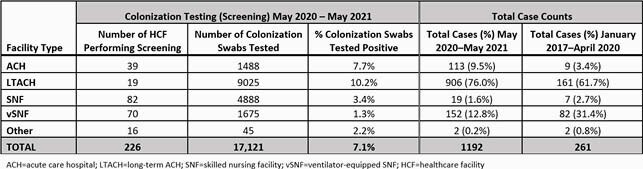

Table 2. COVID-19-related Infection Control Practices Affecting C. auris Spread, and Associated Public Health Recommendations

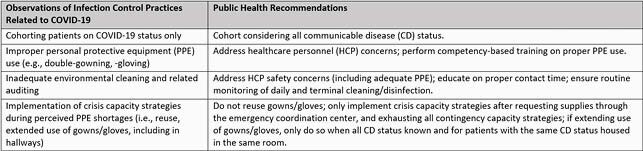

**Conclusion:**

The *C. auris* resurgence in CA was likely a result of COVID-19-related practices and conditions. An aggressive, coordinated, interjurisdictional *C. auris* containment response, including proactive prevention activities at HCF interconnected with outbreak HCF, can help mitigate spread of *C. auris* and potentially other novel AR pathogens.

**Disclosures:**

**All Authors**: No reported disclosures

